# Why Do Some Find it Hard to Disagree? An fMRI Study

**DOI:** 10.3389/fnhum.2015.00718

**Published:** 2016-01-29

**Authors:** Juan F. Domínguez D, Sreyneth A. Taing, Pascal Molenberghs

**Affiliations:** ^1^Social Neuroscience Lab, School of Psychological Sciences and Monash Institute of Cognitive and Clinical Neurosciences, Monash UniversityMelbourne, VIC, Australia; ^2^School of Psychology, University of QueenslandSt Lucia, QLD, Australia

**Keywords:** conflict, individual differences, expertise, cognitive dissonance, social neuroscience, posterior medial frontal cortex, anterior insula

## Abstract

People often find it hard to disagree with others, but how this disposition varies across individuals or how it is influenced by social factors like other people's level of expertise remains little understood. Using functional magnetic resonance imaging (fMRI), we found that activity across a network of brain areas [comprising posterior medial frontal cortex (pMFC), anterior insula (AI), inferior frontal gyrus (IFG), lateral orbitofrontal cortex, and angular gyrus] was modulated by individual differences in the frequency with which participants actively disagreed with statements made by others. Specifically, participants who disagreed less frequently exhibited greater brain activation in these areas when they actually disagreed. Given the role of this network in cognitive dissonance, our results suggest that some participants had more trouble disagreeing due to a heightened cognitive dissonance response. Contrary to expectation, the level of expertise (high or low) had no effect on behavior or brain activity.

## Introduction

The freedom to make autonomous choices (within the limits of the law) without fear of harm or prosecution is a fundamental value at the core of the Universal Declaration of Human Rights, democratic societies and market economies. However, individual choice is never fully autonomous. We may be highly susceptible to the opinions of others (Cialdini and Goldstein, 2004; Levine and Tindale, 2015), which means that our choices are vulnerable to the effects of social influence (Berns et al., 2005). Social influence is not by itself a problem, but it can be deleterious if it occurs unreflectively, as when individuals agree with others for the sake of agreeing. This is potentially a bigger issue than it may first appear given research showing that, without evidence to the contrary, people have a general disposition to believe and agree with others. The deception detection literature, for example, has consistently shown that a percentage of statements judged as truthful is significantly higher than the percentage of statements correctly classified as truthful (see meta-analyses by Vrij, 2000; Bond and DePaulo, 2006). The truth bias, as this phenomenon has been termed, is a necessary social default that facilitates how we interact and deal with other people (Vrij, 2000).

The truth bias, however, represents a challenge for societies to balance. It entails a reduced inclination for individuals to disagree with their peers, with potentially adverse effects on autonomous choice. There is evidence that this reduced inclination to disagree may vary across individuals (Laird and Berglas, 1975; Cialdini et al., 1995; Matz et al., 2008; Campbell-Meiklejohn et al., 2010; Meshi et al., 2012), and that it can be exacerbated by social factors like peer pressure (Berns et al., 2005), and the perceived authority or expertise of others. Expertise, for example, has been found not only to be a powerful source of persuasion (Klucharev et al., 2008) but also to lead to greater agreement (Crano, 1970; Meshi et al., 2012). The impact an inability to critically disagree with others has on everyday situations may be more clearly appreciated when considered in the light of the fallibility of expert advice or imperfect (or even fraudulent) expert pronouncements. For example, advise from financial experts prior to the Global Financial Crisis generated a climate where poor investment decisions were made and sub-prime loans were sought and approved.

Neuroscientists have recently started to investigate the neural mechanisms underlying disagreement. Studies in this area have implicated a set of posterior medial frontal cortex (pMFC) structures [comprising dorsal medial prefrontal cortex, dorsal anterior cingulate cortex (dACC), and pre-supplementary motor area] as well as anterior insula (AI; Westen et al., 2006; Klucharev et al., 2009; Berns et al., 2010; Campbell-Meiklejohn et al., 2010; Shestakova et al., 2013). These areas have previously been found to play a key role in cognitive dissonance (van Veen et al., 2009; Izuma et al., 2010, 2015; Izuma, 2013; Kitayama et al., 2013), a heightened state of mental stress and discomfort experienced by people when their beliefs, ideas or values come into direct contradiction with each other (Festinger, 1957). Consequently, cognitive dissonance is thought to be an important component of the brain's response to disagreement (Berns et al., 2010; Izuma et al., 2015), a view further supported by findings showing that disagreement elicits cognitive dissonance (Festinger, 1957; Matz and Wood, 2005; Matz et al., 2008; Berns et al., 2010). In addition, individual differences in pMFC and AI activity predict attitude change in response to disagreement and counter-attitudinal argumentation, which is suggestive that the resulting cognitive dissonance is more pronounced in some individuals than others (van Veen et al., 2009; Berns et al., 2010). These studies therefore suggest that cognitive dissonance is an important psychological mechanism behind the difficulty some people have to disagree with others and, consequently, the truth bias.

In this fMRI study, we aimed to further explore why people often find it hard to disagree with others, and how this effect is modulated by individual differences and other people's expertise. We were specifically interested in exploring whether cognitive dissonance is a factor in people's disposition to agree/disagree and, consequently, their truth bias. Previous studies have investigated the brain responses to others' subjective opinions that are the same or different from participants' opinions (Klucharev et al., 2009; Berns et al., 2010; Campbell-Meiklejohn et al., 2010; Meshi et al., 2012; Izuma and Adolphs, 2013). As our aim was to assess participants' disposition to disagree rather than their reaction to disagreement, we asked them to make an active decision to agree or disagree with others.

We also wanted to ensure participants' responses reflected their disposition to disagree and the effect of expertise on it, unconfounded by other variables like knowledge of the subject area, personal opinions, valuations or preferences. Therefore, unlike previous studies, the chosen object of disagreement was not subjective opinions but objectively defined statements that could be correct or incorrect. Moreover, the statements were designed to be difficult with the purpose of inducing participants to rely more on the person making the statement and their level of expertise (high vs. low), rather than their own knowledge.

We predicted that, due to the existence of the truth bias, participants would more often agree with the statements than disagree. We expected this would be motivated, at least in part, by the experience of cognitive dissonance during disagreement. Accordingly, we anticipated increased activation in response to disagreement in pMFC and AI. Importantly, individual differences were expected to modulate the above effects as suggested by earlier studies (Campbell-Meiklejohn et al., 2010; Meshi et al., 2012). In line with this, we hypothesized individuals would vary in the extent to which they found it hard to disagree, with some participants being able to disagree with ease (more often) and others only with difficulty (less often). Consequently, we also expected that individuals who disagree less often would show the highest activation in pMFC and AI when they do disagree, consistent with a heightened cognitive dissonance response. In addition, we hypothesized the truth bias would be enhanced by expertise, with participants agreeing more often with people with high vs. low perceived levels of expertise.

## Materials and methods

### Participants

Thirty-nine healthy right-handed participants (19 females; mean age = 22.1 years, *SD* = 5.0 years) took part in this study in exchange for payment. Written informed consent was obtained from each participant in accordance with the Helsinki Declaration. The study was approved by the Behavioral and Social Science Ethical Review Committee of The University of Queensland.

### Experimental stimuli and task

Participants were presented with a total 192 true or false statements from four fields of knowledge: biology (e.g., “Orchid flowers have the most species”), history (e.g., “The first public library was opened in England”), medicine (e.g., “Protanopia is the inability to see the color green”), and physics (e.g., “The faster you move the heavier you get”). For the full list of statements see Supplementary Table 1. In total, each topic contained 24 true and 24 false statements (see Supplementary Figure 1 for distribution of true and false statements across topics). During the experiment, the statements were attributed to an individual with high or low expertise in the subject area: a professor or a student, respectively. There were therefore four types of statements: true statements by a professor (TP), false statements by a professor (FP), true statements by a student (TS), and false statements by a student (FS).

Participants were asked to decide whether the statements were true or false. Agreement was therefore operationalized as true judgments and disagreement as false judgments. Participants were told statements were checked for their accuracy, with many of them found to be incorrect. This was done to ensure participants disagreed (*i.e.*, judged statements as false) often enough. Owing to the existence of the truth bias, it was otherwise expected participants would have a high inclination to agree with a majority of statements. The statements were therefore designed to be difficult.

The difficulty of the statements was confirmed in a prior validation behavioral experiment with an independent cohort of 20 participants (18 females; mean age = 21.6 years, *SD* = 7.0 years). Written informed consent was obtained from each participant in accordance with the Helsinki Declaration. The study was approved by the Behavioral and Social Science Ethical Review Committee of The University of Queensland. Participants were instructed to read each statement carefully and rate how likely they thought each statement is true on a seven-point scale (1 = *I'm sure it is true*, 4 = *I have no idea if it is true or false*, 7 = *I'm sure it is false*). An independent *t*-test revealed that there was no significant difference in truthfulness rating between the true statements (*M* = 4.09, *SD* = 0.54) and false statements (*M* = 4.20, *SD* = 0.50), *t*_(190)_ = −1.41, *p* = 0.160. Participants were therefore, on average, unable to differentiate between true and false statements in the set. Additional independent *t*-tests showed that there was no difference between the number of words for the true (*M* = 8.01, *SD* = 2.07) and false statements (*M* = 7.60, *SD* = 1.97), *t*_(190)_ = 1.43, *p* = 0.155, or between the number of characters for true (*M* = 36.94, *SD* = 9.47) and false statements (*M* = 37.08, *SD* = 9.41), *t*_(190)_ = −0.11, *p* = 0.915.

One half of the true (T1) and one half of the false (F1) statements were randomly assigned to the professors and the other halves of true (T2) and false (F2) statements were assigned to the students. Which subsets (T1, T2, F1, F2) were assigned to professors or students was counterbalanced across participants. A one-way ANOVA revealed no significant differences between the mean truthfulness ratings of any these subsets of statements, *F*_(3, 188)_ = 0.347, *p* = 0.791: T1 (*M* = 4.21, *SD* = 0.41), T2 (*M* = 4.11, *SD* = 0.55), F1 (*M* = 4.15, *SD* = 0.57), and F2 (*M* = 4.11, *SD* = 0.57). There were also no significant differences between the number of words per statement and the number of characters per statement across subsets, *F*_(3, 188)_ = 0.63, *p* = 0.595: T1 (*M* = 37.40, *SD* = 10.44), T2 (*M* = 36.67, *SD* = 9.52), F1 (*M* = 38.50, *SD* = 10.93), and F2 (*M* = 35.90, *SD* = 7.35).

### fMRI paradigm

**Figure 1:**
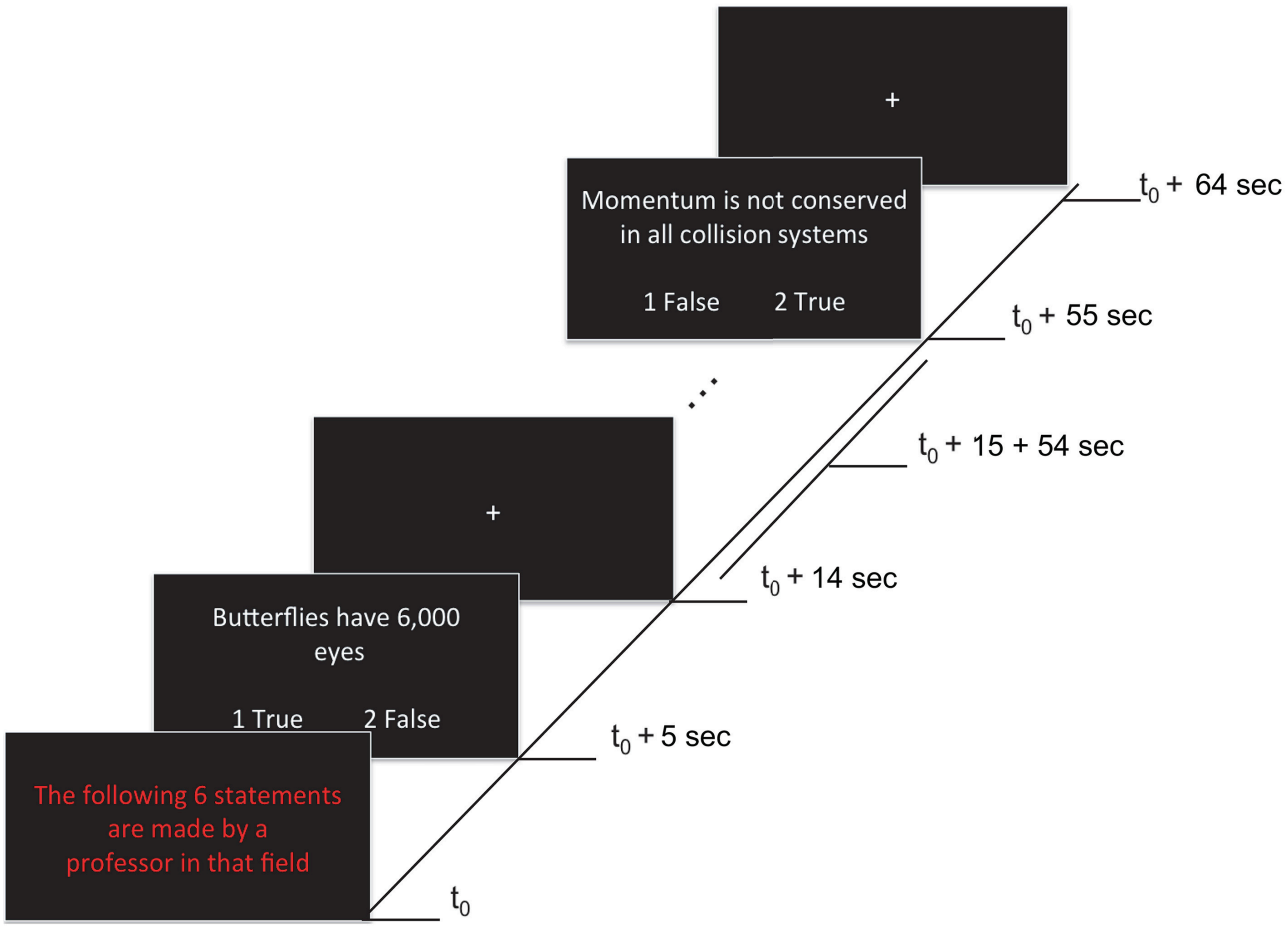
**Stimuli and experimental paradigm**. Each 65 s task block began with a red text indicating the expertise of the person making the statement (5 s). This was followed by six statements (9 s each), each separated by fixation crosses (1 s). Onsets of a sample of events within a block are shown. Responses were made by pressing either button 1 (left) or 2 (right) to indicate True (agreement) or False (disagreement) during the statement presentation. The correspondence between button (1:left, 2:right) and response (true/false) was randomly switched around across statements (as illustrated).

Each of four functional runs contained 12 randomly presented blocks, including eight task blocks (lasting 65 s each) and four rest blocks (lasting 20 s each). Task blocks grouped statements by expertise level: four blocks contained statements attributed to a professor (*i.e*., high expertise level statements) that could be true or false (TP, FP), and the remaining four blocks contained statements attributed to a student (*i.e*., low expertise level statements) that could be true or false (TS, FS). During rest blocks (*i.e.*, baseline condition) a fixation cross was presented at the center of the screen. A fixation cross was also presented for 5 s at the beginning of each run (allowing the magnet to reduce to a steady-state) and for 10 s at the end of each run. A task block started with a 5 s presentation that indicated whether the subsequent six statements were being made either by a professor or a student (*i.e.*, “The following 6 statements are made by a professor in that field”). The statements were presented for 9 s each, followed by a 1 s fixation cross after each statement (see Figure 1). Statements were randomly selected (without replacement) from a list of true and false statements assigned to each level of expertise (see previous section). Each statement type was presented 12 times per run (e.g., 12 TP statements, presented across the four high expertise blocks). In total, each statement type was presented 48 times across the experiment. Participants were given 9 s to read and respond with True or False to each statement using the two-button response pad given. Participants were instructed to use both thumbs when using the response pad. To avoid any confounding effects on neural activation associated with motor responses, the correspondence between button (1:left, 2:right) and response (true/false) was randomly switched around across statements.

### fMRI data acquisition

Structural and functional MR images were acquired with a 3 Tesla scanner (Siemens AG, Erlangen, Germany) and a 32-channel head coil. Functional images were acquired using gradient-echo planar imaging (EPI) with the following parameters: repetition time (TR) = 2.5 s; echo time (TE) = 36 ms; flip angle = 90°; 64 × 64 matrix; voxel size = 3 × 3 × 3 mm. A total of 246 whole brain images per run were acquired, each consisting of 36 transversal slices with 10% gap between each slice. A high-resolution 3D T_1_-weighted image covering the entire brain was also acquired after the second run for anatomical reference (TR = 1900 s; TE = 2.32 ms; flip angle = 9°; 192 × 192 matrix; voxel size = 0.9 × 0.9 × 0.9 mm).

### Data analysis

#### Behavioral data analysis

In this study, there were two factors—agreement and expertise—with two levels each—agree/disagree (corresponding to true/false judgments), and high/low, respectively. This resulted in four conditions: agreement with a professor (AP), disagreement with a professor (FP), agreement with a student (AS), and disagreement with a student (FS). An agreement score was then computed by subtracting disagree (false) from agree (true) scores, collapsed across the levels of expertise (*i.e.*, [DP + DS] − [AP + AS]). A score of 0 indicates that participants disagreed as often as they agreed; a score of -192 means they always agreed; and a score of 192 means they always disagreed. An expertise score was also calculated by determining the difference in scores between professor and student, collapsed across the levels of agreement (*i.e.*, [DP − AP] − [DS − AS]). A score of 0 means that participants trusted the professor as much as the student. Negative scores indicate they believed more in the professor, while positive scores mean they believed more in the student. One sample *t*-tests were then used to evaluate whether the agreement and expertise scores were significantly different from 0. We also conducted a regression analysis of the effect of expertise, agreement and their interaction on response time (RT).

#### fMRI data analysis

Functional MRI data were pre-processed and analyzed with SPM8 Software (Wellcome Department of Imaging Neuroscience, Institute of Neurology, London) through MATLAB (MathWorks Inc., USA). All EPI images were first realigned to the first image of each run to offset any effects of head movements. The T_1_-weighted anatomical scan for each participant was then coregistered to the mean functional image created during realignment. Using the segment routine, the coregistered anatomical scan was then normalized with a voxel size of 1 × 1 × 1 mm to the MNI T_1_ standard template (Montreal Neuropsychological Institute). Following this, the same parameters were used to normalize all of the EPI images to map onto the template using a voxel size of 3 × 3 × 3 mm. The images were then smoothed with a 9-mm isotropic Gaussian kernel.

A general linear model was used to estimate regions of significant Blood Oxygen Level Dependent (BOLD) response in each voxel for each participant and included event-related regressors for each of four conditions (AP, DP, AS, and DS); that is, each condition was modeled based on the participant's agree/disagree (true/false) responses to the statements. The onset of each event corresponded to the start of the slides in which the statements were shown and had a duration of 9 s. Contrast images for each participant across all conditions were included at the second level in a 2 (agreement: Agree vs. Disagree) × 2 (expertise: Professor vs. Student) factorial design, and the main effects and interaction effects were estimated. The above experimental design can therefore be effectively conceived as mixed, since statements were blocked by level of expertise, but could only be sorted by agreement *post-hoc*. Thus, the block aspect of the study aimed to increase the design efficiency with respect to expertise. Being response-dependent, the agreement factor had a random distribution of event types (agree vs. disagree), which ensured that the design had an adequate level of efficiency with respect to this factor (Dale, 1999; Friston et al., 1999).

To investigate if participants who disagreed less often had more activation in the pMFC and AI when they disagreed, we modeled the relationship between the BOLD response exclusive to the disagreement compared to agreement condition (*i.e.*, the Disagree minus Agree contrast), with the agreement score (*i.e*., [DP + DS] − [AP + AS]). Specifically, we expected a negative correlation between the two. That is, the less people disagree (*i.e*., the lower the agreement score), the more activation we expected in pMFC and AI when people disagree (*i.e*., the Disagree minus Agree contrast). A cluster-level threshold with family-wise error rate (FWE) of *p* < 0.05 corrected for the whole brain was used to identify significant results for all analyses, with clusters defined by a voxel level threshold of *p* < 0.001.

## Results

### Behavioral results

The agreement score was not significantly different from 0 (*M* = 0.64, *SD* = 53.84), *t*_(38)_ = 0.07, *p* = 0.941. Similarly, the expertise score was not significantly different from 0 (*M* = −2.13, *SD* = 19.58), *t*_(38)_ = −0.68, *p* = 0.493 (The expertise score was not normally distributed so a bootstrapped intercept-only regression model with 5000 iterations was used). No significant effect of agreement (*z* = 0.13, *p* = 0.89), expertise (*z* = 0.20, *p* = 0.84) or their interaction (*z* = −0.12, *p* = 0.91) was found on RT (results from a bootstrapped regression with 5000 iterations are reported as normality assumptions were not met; AP RT *M* = 4286 ms, *SD* = 781 ms; DP RT *M* = 4309 ms, *SD* = 752 ms; AS RT *M* = 4321 ms, *SD* = 769 ms; and DS RT *M* = 4316 ms, *SD* = 734 ms).

### fMRI results

**Figure 2:**
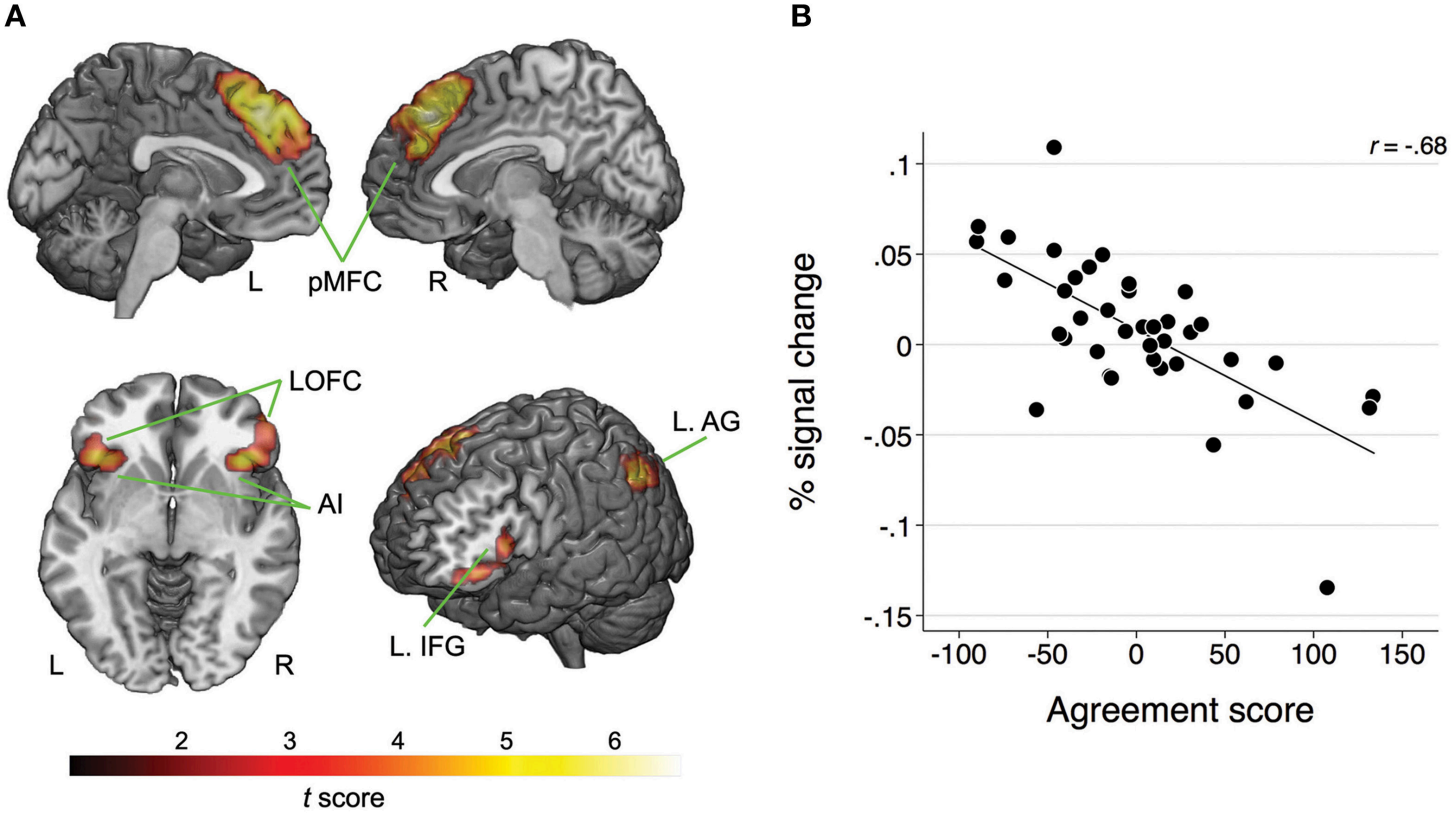
**(A)** Map of clusters showing a significant association between the BOLD response and the agreement scores for the Disagree minus Agree contrast; pMFC, posterior medial frontal cortex; AI, anterior insula; IFG, inferior frontal gyrus; LOFC, lateral orbitofrontal cortex; AG, angular gyrus; L, left; R, right. For MNI coordinates of peak voxels and statistics refer to Table 1. **(B)** Scatterplot and fitted line illustrating the negative relationship between the BOLD response (averaged across all clusters) and agreement scores for the Disagree minus Agree contrast. Lower scores on the agreement score indicate that the participants were less likely to disagree.

Whole-brain analysis revealed no main effect of agreement or expertise. There was also no interaction between the two factors. However, there was a significant association between BOLD response and the agreement score exclusive to the Disagree minus Agree contrast in four clusters comprising the following network: one cluster corresponded to pMFC bilaterally, specifically comprising dorsal medial prefrontal frontal cortex (dMPFC), dACC, and pre-supplementary motor area (pre-SMA); a second cluster with peak voxel on lateral orbitofrontal cortex (LOFC) also included AI on the right; another cluster with peak voxel on LOFC incorporated AI and inferior frontal gyrus (IFG); one last cluster involved the left angular gyrus (AG; see Table 1 and Figure 2A). We averaged the percentage signal change across all voxels of the four clusters for each participant using MarsBaR (http://marsbar.sourceforge.net) and performed a correlation analysis with the agreement score to better illustrate the direction of the relationship and its strength. We found a strong negative correlation (*r* = −0.68) between these variables. The less often participants disagreed, the more activation they showed in the network of relevant brain areas when they did disagree (see Figure 2B).

**Table 1:** **Clusters exhibiting a significant association between BOLD response and the agreement score for the Disagree minus Agree contrast (FWE-corrected)**.

**Anatomical extent of clusters**	**Cluster size**	**MNI coordinates of peak voxel**				***t*-score**	***p*-value**
		**Side**	**x**	**y**	**z**		
pMFC bilaterally	1182	B	0	35	43	6.58	< 0.001
AI/IFG/OFC	217	L	−42	23	−5	4.43	0.033
AI/OFC	211	R	51	41	−8	6.01	0.035
AG	309	L	−45	−64	49	4.59	0.012

pMFC, posterior medial frontal cortex; AI, anterior insula; IFG, inferior frontal gyrus; OFC, orbitofrontal cortex; AG, angular gyrus; L, left; R, right; B, bilateral.

### Results from additional analyses

We carried out an additional analysis as a test of whether a low proportion of disagreement truly reflects a difficulty to disagree. First, we calculated the relative agreement RT (*i.e.*, Disagree RT minus Agree RT) for each participant. We then correlated this measure with the agreement score. We found there was a significant negative correlation, ρ = −0.49, *p* = 0.001 (Spearman's ρ is reported as normality assumptions were violated; Pearson's correlation, *r* = −0.55, was also significant at *p* < 0.001).

To rule out potential confounding effects on the results, a number of additional analyses were performed. First, participants' responses could have been biased by the truth-like quality of statements (*i.e*., the extent to which false statements, by virtue of being counterfeit, were intuitively perceived as less truthful). To assess this possibility, we calculated the average proportion of agreement with each of the four type of statements (n/48): TP = 0.54 (*SD* = 0.16); FP = 0.47 (*SD* = 0.16); TS = 0.52 (*SD* = 0.15); FS = 0.47 (*SD* = 0.16). The proportion of agreement with true statements (TP, TS; *M* = 0.53, *SD* = 0.14) was statistically higher than the proportion of agreement with false statements (FP, FS; *M* = 0.47, *SD* = 0.15), as revealed by a paired *t*-test: *t*_(38)_ = 4.79, *p* < 0.001. This indicates that participants may have been biased by the truth-like quality of statements. We then calculated a difference score between the proportion of agreement with false vs. true statements and evaluated its effect over the agreement score (with a regression model) and the BOLD response for the Disagree minus Agree contrast (with a whole brain voxel-wise analysis that modeled the relationship between the BOLD response for the Disagree minus Agree contrast, with the difference score between the proportion of agreement with false vs. true statements). The absence of a significant effect in both cases would be supportive of a bias due to the truth-like quality of statements not influencing people's disposition to agree or the brain response to disagreement. We found no significant effect over the agreement score (*t*_(38)_ = −0.66, *p* > 0.05) or the BOLD response for the Disagree minus Agree contrast (*p* > 0.05, cluster corrected).

The pattern of brain activity we found could have also been influenced by a salience effect stemming from a low proportion of instances of disagreement for participants who disagreed less often. To address this possibility, we estimated the proportion of trials where participants who disagreed less often (defined stringently as the bottom 25th percentile on the agreement score) actually disagreed. We found these participants (*n* = 10) disagreed on average 65.7 trials out of 192 (range 50–76) or 34% of the time.

## Discussion

In this study, we used fMRI to ascertain why people often have an aversion to disagree with others, and how this effect is modulated by individual differences and other people's expertise. We therefore asked participants, for the first time, to make the active decision to agree or disagree with others. We also ensured that this decision reflected participants' disposition to disagree unconfounded by other factors. Results confirm our prediction that individual differences modulate a brain network previously involved in disagreement and cognitive dissonance comprised of posterior medial prefrontal cortex (pMFC) structures and AI. Specifically, participants who disagreed less frequently exhibited greater activation in this network when they actually disagreed.

Both pMPC and AI have been consistently found to respond to disagreement (Klucharev et al., 2009; Berns et al., 2010; Campbell-Meiklejohn et al., 2010). These areas are also directly engaged by cognitive dissonance in the context of counter-attitudinal behavior (van Veen et al., 2009; Izuma et al., 2010). The involvement of these brain areas in the present study therefore suggests that actually making the decision to disagree elicited greater cognitive dissonance in those individuals who disagreed less often. This may explain why these individuals found it more difficult to disagree, as they did so only infrequently: they experienced a heightened state of mental stress and discomfort, which they would tend to avoid. Consistent with the interpretation that some participants found it more difficult to disagree is the negative correlation we found between relative agreement RT and the agreement score. This indicates that those who disagreed less often were slower to respond when they disagreed relative to when they agreed.

Broadly speaking, pMFC is involved in on-line monitoring and control of action, which is especially important in situations involving conflict between alternatives. In particular, this area plays a key role in reinforcement learning (in both social and non-social contexts, Ruff and Fehr, 2014), by detecting conflict between actual and predicted states resulting in a prediction-error signal that can be used for subsequent adjustment of behavior (Schultz and Dickinson, 2000; Holroyd and Coles, 2002). There is evidence that aversive social experiences like disagreement (Klucharev et al., 2009; Berns et al., 2010; Campbell-Meiklejohn et al., 2010; Izuma and Adolphs, 2013) or agreeing with disliked people (Izuma and Adolphs, 2013) share similar mechanisms with reinforcement learning (Izuma and Adolphs, 2013; Izuma et al., 2015). Tellingly, transcranial magnetic stimulation to pMFC has been found to reduce social conformity (Klucharev et al., 2011), as well as preference change after cognitive dissonance (Izuma et al., 2015). This suggests the pMFC plays a causal role in behaviors that require conflict monitoring. Not only does conflict during these circumstances trigger a neural response in pMFC, but this signal is also predictive of subsequent behavioral adjustments, suggesting resolution of dissonance (Klucharev et al., 2009; Berns et al., 2010; Campbell-Meiklejohn et al., 2010; Izuma and Adolphs, 2013). In the present study, the activation of pMFC in those participants who disagreed less often is therefore consistent with the triggering of a conflict detection signal.

As in previous studies involving disagreement (Klucharev et al., 2009; Berns et al., 2010; Campbell-Meiklejohn et al., 2010; Izuma and Adolphs, 2013), the pMFC activation included also dACC and pre-SMA. Dorsal ACC is being increasingly recognized as a node that integrates relevant information to control action motivated by negative affect or aversive stimuli under uncertainty, as when there is competition between plausible alternatives (Shackman et al., 2011). Pre-SMA, in turn, is involved in linking cognition and action, accordingly integrating prefrontal and motor systems (Nachev et al., 2007, 2008). Specifically, pre-SMA has been implicated in the resolution of competition between action contingencies (Nachev et al., 2007; Izuma and Adolphs, 2013). Dorsal ACC and pre-SMA involvement in the current study is therefore in agreement with these areas playing a supporting role in the handling and resolution of conflict.

AI is thought to be involved in cognitive dissonance by virtue of its role in negative affect and autonomic arousal (van Veen et al., 2009; Kitayama et al., 2013). Conflict resulting from disagreement could therefore be expected to lead to increased negative affect and the associated activation in AI. However, rather than simple involvement in affect, mounting evidence indicates that AI has an integrative function, linking affective with interoceptive and cognitive information. As a result, AI is thought to generate emotional awareness, a unified meta-representation of global emotional moments available to consciousness (for reviews see Kurth et al., 2010; Lamm and Singer, 2010; Gu et al., 2013; see also, Craig, 2010).

A recent model postulates AI performs a more general function as an internal hub in salience detection (Menon and Uddin, 2010). In this model, AI mediates dynamic interactions between large-scale networks involved in externally and internally oriented processes leading to a heightened state of emotional awareness with respect to a salient event. The sense of contradiction and inconsistency that characterizes cognitive dissonance can therefore be thought of as being intrinsically salient and is therefore likely to be experienced as a heightened awareness of an emotional state.

Together with pMFC and AI, a number of other areas, including IFG and angular gyrus (AG) in the left hemisphere, as well as lateral orbitofrontal cortex (LOFC) bilaterally, were active in those who disagreed less often. IFG (Campbell-Meiklejohn et al., 2010) and LOFC (Campbell-Meiklejohn et al., 2010; Meshi et al., 2012) activity has previously been reported in the context of disagreement. IFG and AG have also been implicated in cognitive dissonance arising from counter-attitudinal conflict (van Veen et al., 2009; Izuma et al., 2010).

More broadly, IFG and AG have been shown to play a role in inhibition of prepotent responses (Aron et al., 2004; Molenberghs et al., 2009; Seghier, 2013), which in the present study may represent an inhibitory response to agreeing (the prepotent response) when the actual response was disagreeing. Consequently, these areas could be directly assisting pMFC's cognitive dissonance response arising from disagreement in terms of response to conflict.

LOFC has an important function in the representation of displeasure (Kringelbach and Rolls, 2004; Hayes and Northoff, 2011) and the anticipation of negative outcomes (Amodio and Frith, 2006; Ursu et al., 2008). The involvement of this area in the present study is therefore consistent with the mental stress and discomfort characteristic of cognitive dissonance potentially being experienced by those participants who disagreed infrequently.

Previous studies have reported individual brain activity differences in the network reported in the present study (including pMFC, AI, IFC, LOFC), depending on how much participants were influenced by others' opinions (Berns et al., 2010; Campbell-Meiklejohn et al., 2010; Meshi et al., 2012). Influence was measured in terms of the magnitude of the difference of opinion (*i.e.*, disagreement). This further reinforces the view that these structures form part of a network involved in a cognitive dissonance response arising from disagreement (Berns et al., 2010), and that they are subject to individual differences. However, these studies have had a reactive rather than a proactive focus: they have investigated participants' reactions to opinions or judgments that disagree with their own, rather than participants' active decision to disagree. Moreover, these previous studies used differences in subjective preferences or valuations, rather than difficult objective facts. The manipulations we introduced in this study effectively allowed us to measure participants' disposition to disagree unconfounded by factors like preferences, valuations, and knowledge. Our results therefore extend previous findings by indicating that the same network of structures is involved in disagreement whether it is reactive or proactive.

Our agreement score can be interpreted as a measure of the disposition to disagree (or agree), effectively a proxy for the truth bias, and may be considered a personality trait encoded in this network's response. This was explicitly anticipated in the original formulation of cognitive dissonance (Festinger, 1957), where personality differences were expected with respect to “fear of dissonance” and peoples' capacity to reduce dissonance. In support of this view, subsequent studies have reported a number of personality traits to be (positively or negatively) associated with the cognitive dissonance response. These traits include extraversion (Matz et al., 2008), preference for consistency (Cialdini et al., 1995), self-attribution (Laird and Berglas, 1975) as well as psychopathic personality traits (Murray et al., 2012).

Confounding factors could have potentially affected our results. First, participant's responses could have been biased by the truth-like quality of the statements. While the proportion of agreement with true statements was statistically higher than the proportion of agreement with false statements, we found that the difference between the proportion of agreement with false vs. true statements had no effect on either the agreement score or the brain response to disagreement. This indicates that the truth-like quality of the statements had no effect on our results. Second, rather than greater difficulty to disagree for those who disagreed less, the pattern of brain activations we found could reflect higher salience associated with a low proportion of instances of disagreement for participants who disagreed less often. Contradicting this possibility, participants who disagreed less often still disagreed a sizeable ~35% of the time.

Conflicting with our predictions, only half of participants agreed more often with the statements. Therefore, we found overall no evidence of an agreement effect or truth bias (Vrij, 2000). In addition, contrary to expectation, participants did not agree more often with people with high levels of expertise. Consistent with these behavioral results, there was also no significant brain activation associated with the main effects of agreement or expertise, or their interaction. The absence of an agreement effect, behavioral or neural, could be due to the fact that participants may have found the experiment artificial, as they were told that many statements were false and to actively look out for them. Therefore, participants may have had a high motivation to disagree. That said, the proportion of statements agreed or disagreed with, varied widely across participants (as is apparent in Figure 2B). This may have canceled out the effect of the truth bias (while at the same time accentuating individual differences). Another explanation could be that the experiment was low stake and, as there were no immediate or adverse ramifications, participants may have felt there were no impediments to disagreeing.

The failure to observe an expertise effect, on behavior or brain activity, may be related to a potentially weak priming of the expertise condition, with expertise priming presented only briefly (during 5 s) at the beginning of each (65 s) block. Alternatively, there may have been a group membership confound. In particular, a student in-group bias may have canceled out the effect of the high expertise group—on account of favoritism for one's in-group (Crocker and Luhtanen, 1990; Yamagishi et al., 1997; Molenberghs, 2013)—as participants were mostly university students (~95%).

A small number of studies have investigated disagreement behavior and its neural basis, together with how behavior and brain responses vary across individuals and in response to social factors (Klucharev et al., 2009; Berns et al., 2010; Campbell-Meiklejohn et al., 2010; Meshi et al., 2012). However, this study is the first attempting to evaluate peoples' disposition to disagree independent from prior knowledge, opinion, preferences or valuations, and the effect that a social factor (expertise) may have on this disposition. For this reason, our results are the most direct evidence of a network responsive to disagreement that reflects individual differences in people's disposition to disagree. Moreover, the structures that comprise this network suggest cognitive dissonance is a potential mechanism explaining why some individuals may be motivated to avoid disagreement. Overall, the results suggest that disagreement triggers a cognitive dissonance response where neural resources are deployed to handle increased conflict and negative arousal, and that this response varies across individuals.

Having a lot of trouble disagreeing due to a heightened cognitive dissonance response may be indicative of an array of emotional, attitudinal or social issues compromising an individual's capacity to make autonomous choices. This can potentially lead to poor decision-making, anxiety, or difficulties in interpersonal relationships. For example, introversion has been shown to be associated with the experience of greater dissonance discomfort (Matz et al., 2008). Moreover, evidence indicates sensitivity to dissonance is linked to increased susceptibility to influence (Berns et al., 2010; Campbell-Meiklejohn et al., 2010; Meshi et al., 2012). An excessive, pathological aversion to cognitive dissonance has even been envisioned, where individuals would effectively exhibit an inability to make decisions, on account of extreme avoidance of conflicting views and change in behavior (Festinger, 1957). Problems such as these would need to be addressed by individuals, researchers, and health practitioners alike. The results presented here provide a better understanding of the neural and psychological mechanisms involved in disagreement and can potentially be used to design better-targeted and effective interventions in the future.

## Funding

This work was supported by an Australian Research Council (ARC) Early Career Research Award (DE130100120), Heart Foundation Future Leader Fellowship (100458), and ARC Discovery Grant (DP130100559) awarded to PM.

### Conflict of interest statement

The authors declare that the research was conducted in the absence of any commercial or financial relationships that could be construed as a potential conflict of interest.
