# Supplementary material for: Why Do Some Find it Hard to Disagree? An fMRI Study
Source: Front Hum Neurosci. 2016 Jan 29;9:718. doi: 10.3389/fnhum.2015.00718 (PMC4731490; doi:10.3389/fnhum.2015.00718)
Supplement: Supplementary file 1 [file Table1.DOCX]

| **Supplementary Table 1. Appendix**  List of true and false statements  Includes word and character count, as well as mean truthfulness rating. | | | | | | | | | |  |
| --- | --- | --- | --- | --- | --- | --- | --- | --- | --- | --- |
| **Statement** | | **Truthfulness** | | **Words** | | **Chars** | | **Rating** | |  |
| 15% of American males are color blind | | True | | 7 | | 32 | | 4.5 | |  |
| 90% of bird species are monogamous | | True | | 6 | | 29 | | 4 | |  |
| A columella is the space between your nostrils | | True | | 8 | | 39 | | 4.2 | |  |
| A corrugator is located on your forehead | | True | | 7 | | 34 | | 4.35 | |  |
| A haemodializar is a mechanical lung | | False | | 6 | | 31 | | 3.75 | |  |
| A loss of alpha particles increases the atomic number | | False | | 9 | | 45 | | 3.5 | |  |
| A lurcher is a type of cat | | False | | 7 | | 20 | | 4.3 | |  |
| A mouse’s sperm is bigger than an elephant’s | | True | | 8 | | 37 | | 4.4 | |  |
| A patella is part of your knee | | True | | 7 | | 24 | | 4.4 | |  |
| A prism produces a rainbow from white light by concentration | | False | | 10 | | 51 | | 3 | |  |
| A tittiliomaniac has a compulsion to scratch | | True | | 7 | | 38 | | 3.4 | |  |
| Ageusia is the loss of taste | | True | | 6 | | 23 | | 3.9 | |  |
| Amazon river dolphins are green in color | | False | | 7 | | 35 | | 4.3 | |  |
| An alopecia sufferer lacks hair | | True | | 5 | | 27 | | 4.5 | |  |
| Ancient Egyptians shaved their eyebrows when their cats died | | True | | 9 | | 52 | | 3.4 | |  |
| Ants always fall on their left side when drunk | | False | | 9 | | 38 | | 4.15 | |  |
| Ants live in a formicarium | | True | | 5 | | 22 | | 5 | |  |
| Armadillos do not have any teeth | | True | | 6 | | 27 | | 3.9 | |  |
| Astronaunts cannot burp in space | | True | | 5 | | 28 | | 4.35 | |  |
| At 100 degrees, steam and liquid water molecules have different kinetic energies | | False | | 12 | | 69 | | 4.2 | |  |
| At least a quarter of humanity is short sighted | | True | | 9 | | 39 | | 3.35 | |  |
| Atephobia is the fear of perfection | | False | | 6 | | 30 | | 3.95 | |  |
| Australian women won the right to vote in 1902 | | True | | 9 | | 38 | | 4.5 | |  |
| Babies are born without knee caps | | True | | 6 | | 26 | | 3.85 | |  |
| Bats always turn right when exiting a cave | | False | | 8 | | 35 | | 3.8 | |  |
| Bats have the lowest male homosexuality rate of all mammals | | False | | 10 | | 50 | | 4.6 | |  |
| Bears can run as fast as dogs | | False | | 7 | | 23 | | 4.05 | |  |
| Bedbugs can live up to two years without food | | False | | 9 | | 37 | | 4.65 | |  |
| Beijing was the first city to reach one million in population | | False | | 11 | | 51 | | 3.9 | |  |
|  | |  | |  | |  | |  | |  |
| **Statement** | | **Truthfulness** | | **Words** | | **Chars** | | **Rating** | |  |
| Beriberi is a condition caused by a lack of vitamin B1 | | True | | 11 | | 27 | | 3.65 | |  |
| Beta particles are the same as helium nuclei | | False | | 8 | | 37 | | 3.35 | |  |
| Black holes are not black | | True | | 5 | | 21 | | 4.4 | |  |
| Black holes exist at the corner of every galaxy | | False | | 9 | | 39 | | 3 | |  |
| British spies once used urine as invisible ink | | False | | 8 | | 39 | | 3.9 | |  |
| Brown headed men are more likely to go bald | | False | | 9 | | 35 | | 4 | |  |
| Butterflies have 6,000 eyes | | False | | 4 | | 24 | | 5 | |  |
| Caterpillars have approximately 124 muscles in its head | | False | | 8 | | 48 | | 5 | |  |
| Cats were the symbol of freedom in ancient Rome | | True | | 9 | | 39 | | 4.15 | |  |
| Checkers is older than chess | | True | | 5 | | 32 | | 4.25 | |  |
| Cleopatra was the first in her family to learn Egyptian | | True | | 10 | | 46 | | 4.15 | |  |
| Cockroaches fart every seven minutes | | False | | 5 | | 32 | | 3.8 | |  |
| Cockroaches will eat anything except for carrots | | False | | 7 | | 42 | | 4.2 | |  |
| Coherent light waves are all in phase | | True | | 7 | | 31 | | 4.65 | |  |
| Cold water freezes faster than hot water | | False | | 7 | | 34 | | 5 | |  |
| Cornflakes was invented in 1790 | | False | | 5 | | 27 | | 3.75 | |  |
| Cretinism is caused by a failure of the pineal gland | | False | | 10 | | 43 | | 3.1 | |  |
| Disk brakes was invented in 1952 | | False | | 6 | | 27 | | 4.3 | |  |
| Dolphins sleep with one eye open | | True | | 6 | | 27 | | 3.9 | |  |
| Dragonflies have the best eyesight of the insect family | | True | | 9 | | 47 | | 3.75 | |  |
| During menstruation, the sensitivity of a woman’s pinkie finger is reduced | | False | | 1 | | 64 | | 4.5 | |  |
| Ecchymosis is another name for a fracture | | False | | 7 | | 35 | | 3.45 | |  |
| Electromagnetic waves originate from accelerating charged particles | | True | | 7 | | 61 | | 4.9 | |  |
| Elephants are the only mammal that cannot jump | | True | | 8 | | 39 | | 4.35 | |  |
| Elephants have 20,000 muscles in its trunk | | False | | 7 | | 36 | | 3.25 | |  |
| Emus are the only birds that can smell | | True | | 8 | | 31 | | 4.15 | |  |
| Energy is condensed matter | | True | | 4 | | 23 | | 4 | |  |
| England was the first country that allowed women to vote | | False | | 10 | | 47 | | 5.05 | |  |
| Epistaxis is another name for coughing up blood | | True | | 8 | | 40 | | 3.65 | |  |
| Every second around 1,000 lightning bolts strike the Earth | | False | | 9 | | 50 | | 4.3 | |  |
| **Statement** | | **Truthfulness** | | **Words** | | **Chars** | | **Rating** |  |  |
| Feet contain the most sweat glands | | True | | 6 | | 29 | | 4.05 |  |  |
| Female pigeons cannot lay an egg unless she sees another pigeon | | True | | 11 | | 30 | | 4.4 |  |  |
| Fingernails contain the most gold in the human body | | False | | 9 | | 43 | | 4.9 |  |  |
| Flushable toilets were in use in ancient Rome | | True | | 8 | | 38 | | 4.55 |  |  |
| Geiger counters are used to detect or observe radiation | | True | | 9 | | 36 | | 4.5 |  |  |
| Giraffes are the only creature born with horns | | True | | 8 | | 39 | | 4.05 |  |  |
| Goats are the only animals with rectangular pupils | | False | | 8 | | 43 | | 3 |  |  |
| Gorillas are excellent swimmers | | False | | 4 | | 28 | | 4.85 |  |  |
| Guns were invented in China | | True | | 5 | | 23 | | 4.75 |  |  |
| Hippophagic societies support eating horsemeat | | True | | 5 | | 42 | | 5 |  |  |
| Humans are the only animal that sleep on their back | | False | | 10 | | 42 | | 4.15 |  |  |
| Hydrosis is the medical term for shivering | | False | | 7 | | 36 | | 3.75 |  |  |
| Hypermetropic people are short sighted | | False | | 5 | | 34 | | 4.75 |  |  |
| Ictheologist is the study of fish | | True | | 6 | | 28 | | 4.45 |  |  |
| In 1800’s, the population of the world reached one billion | | True | | 10 | | 49 | | 4.25 |  |  |
| In 1820, Bachelors were taxed in Missouri | | True | | 7 | | 31 | | 3.75 |  |  |
| Increasing light intensity decreases the emission of photo-electrons | | False | | 8 | | 61 | | 4.7 |  |  |
| Insulators contain free electrons | | False | | 4 | | 30 | | 4.4 |  |  |
| Japanese sea squirts eat their own brain | | True | | 7 | | 34 | | 4.55 |  |  |
| Killer whales can make the loudest noise of all animals | | False | | 10 | | 46 | | 3.75 |  |  |
| Kissing was once illegal in China | | False | | 6 | | 28 | | 3.65 |  |  |
| Kleenex tissues were used as gas mask filters in WW1 | | True | | 10 | | 33 | | 4.55 |  |  |
| Large objects have very long wavelengths | | False | | 6 | | 35 | | 4.65 |  |  |
| Leaches have a total of 32 brains | | True | | 7 | | 27 | | 5.4 |  |  |
| Light does not always travel very fast | | True | | 7 | | 32 | | 5.6 |  |  |
| Light waves are longitudinal and mechanical | | False | | 6 | | 38 | | 4 |  |  |
| Lightning bolts are half as hot as the sun | | False | | 9 | | 34 | | 4.4 |  |  |
| Lions have killed more people in Africa than any other animal | | False | | 11 | | 51 | | 5.45 |  |  |
| Long fat cold wires make the best conductors | | False | | 8 | | 37 | | 4.55 |  |  |
| Louis Bleriot piloted the first flight across the English channel | | True | | 10 | | 56 | | 4.05 |  |  |
| Magnifying glass is always made out of convex lens | | True | | 9 | | 42 | | 3.55 |  |  |
| **Statement** | | **Truthfulness** | | **Words** | | **Chars** | | **Rating** |  |  |
| Mass and inertia are the same thing | | True | | 7 | | 29 | | 5.3 |  |  |
| Men with chest hair are more likely to get cirrhosis | | False | | 10 | | 43 | | 4.65 |  |  |
| Millikan determined the charge on a single electron | | True | | 8 | | 44 | | 3.9 |  |  |
| Momentum is not conserved in all collision systems | | False | | 8 | | 43 | | 4.35 |  |  |
| Monochromatic lights have one frequency | | True | | 5 | | 35 | | 3.35 |  |  |
| Most giraffes are bisexual | | True | | 4 | | 23 | | 4.55 |  |  |
| Napoleon Bonaparte died in St Margereta | | False | | 6 | | 34 | | 3.7 |  |  |
| \| Only charged particles can be accelerated in a particle accelerator \| True \| 10 \| 58 \| 3.55 \| \| --- \| --- \| --- \| --- \| --- \|   Orchid flowers have the most species | | True | | 6 | | 28 | | 3.9 |  |  |
| Our eyelashes are replaced every three months | | True | | 7 | | 39 | | 4.3 |  |  |
| Paludism is an old name for malaria | | True | | 7 | | 25 | | 4.1 |  |  |
| Paper originated from Rome | | False | | 4 | | 23 | | 3.1 |  |  |
| People are better at judging how an object fall when standing | | False | | 11 | | 51 | | 4.05 |  |  |
| People have been wearing glasses for around 700 years | | True | | 9 | | 45 | | 4.2 |  |  |
| People weigh slightly less when the moon is directly overhead | | True | | 10 | | 52 | | 3.9 |  |  |
| Percoids is a type of bony fish | | True | | 7 | | 25 | | 3.05 |  |  |
| Phase change are due to kinetic energy changes | | False | | 8 | | 39 | | 4.2 |  |  |
| Pinkie nails grow the fastest | | False | | 5 | | 25 | | 3.7 |  |  |
| Planets have magnetic field due to their liquid iron core | | True | | 10 | | 48 | | 4.6 |  |  |
| Pogonophobia is the fear of hair | | False | | 6 | | 27 | | 4.25 |  |  |
| Ponytails were once banned in China | | False | | 6 | | 30 | | 5.3 |  |  |
| Proportionately, ants have the largest brain of all animals | | True | | 9 | | 51 | | 4.45 |  |  |
| Protanopia is the inability to see the color green | | False | | 9 | | 43 | | 3.2 |  |  |
| Pumice is a rock that floats in water | | True | | 8 | | 30 | | 4.6 |  |  |
| Pyramids were built by paid labourers | | True | | 6 | | 32 | | 4.05 |  |  |
| Quinsy is the inflammation of the throat | | False | | 7 | | 34 | | 3.55 |  |  |
| Radioactive half-lives cannot be changed by heat or pressure | | True | | 9 | | 52 | | 4.75 |  |  |
| Radiowaves travel at the speed of light | | True | | 7 | | 34 | | 4.8 |  |  |
| Rats and horses are the only animals that cannot vomit | | True | | 10 | | 45 | | 4 |  |  |
| Rickets is caused by a lack of sodium in the diet | | False | | 11 | | 39 | | 4.85 |  |  |
| Scallops have 15 blue eyes | | False | | 5 | | 22 | | 4.05 |  |  |
| Scotopic people can see in the dark | | True | | 7 | | 29 | | 3.5 |  |  |
|  | |  | |  | |  | |  |  |  |
| **Statement** | | **Truthfulness** | | **Words** | | **Chars** | | **Rating** |  |  |
| Sea otters eat and sleep on their back | | True | | 8 | | 31 | | 4.45 |  |  |
| Seven of the 10 deadliest wars were in Rome | | False | | 9 | | 35 | | 4.2 |  |  |
| Sharks are the only fish that can blink with both eyes at the same time | | True | | 15 | | 57 | | 3.8 |  |  |
| Sliced bread was introduced in 1878 | | False | | 6 | | 30 | | 4.2 |  |  |
| Slugs have a total of four noses | | True | | 7 | | 26 | | 4.45 |  |  |
| Snails can sleep for up to one year | | False | | 8 | | 28 | | 4.5 |  |  |
| Snakes can have more than one lung | | False | | 7 | | 28 | | 3.75 |  |  |
| Solar flares have the energy of 1,000 atom bombs | | False | | 9 | | 40 | | 4 |  |  |
| Sony invented the transistor radio | | True | | 5 | | 30 | | 3.8 |  |  |
| Sound waves are transverse | | False | | 4 | | 23 | | 3.65 |  |  |
| Starfish are the only creature that can turn its stomach inside out | | True | | 12 | | 56 | | 4.4 |  |  |
| Stars twinkle because of the wafting of the atmosphere | | True | | 9 | | 35 | | 3.45 |  |  |
| Sunlight exerts no pressure | | False | | 4 | | 24 | | 4.7 |  |  |
| Syncope is the medical name for nausea | | False | | 7 | | 32 | | 4.3 |  |  |
| The driest substance in the world is polytetrafluoroethene | | False | | 8 | | 51 | | 4.45 |  |  |
| The average person spends eight years of their life being ill | | True | | 11 | | 51 | | 4.2 |  |  |
| The battle of the Atlantic was the shortest battle of WWII | | False | | 11 | | 48 | | 4.05 |  |  |
| The catfish has the most taste buds of all animals | | True | | 10 | | 41 | | 3.6 |  |  |
| The discoid cockroach is the fastest insect in the world | | False | | 10 | | 47 | | 4.8 |  |  |
| The dollar sign was invented in 1878 | | False | | 7 | | 30 | | 5 |  |  |
| The Escorial palace in Spain had 1,200 doors | | True | | 8 | | 37 | | 4.3 |  |  |
| The face is the dirtiest skin on the human body | | True | | 10 | | 38 | | 4.3 |  |  |
| The faster you move, the heavier you get | | True | | 8 | | 33 | | 4.75 |  |  |
| The fastest speed a falling raindrop can travel is 29km/h | | True | | 10 | | 48 | | 3.8 |  |  |
| The fastest swimming marine mammal is the blue whale | | False | | 9 | | 44 | | 3.7 |  |  |
| The first false teeth were made from ivory | | True | | 8 | | 35 | | 3.8 |  |  |
| The first public library was opened in England | | False | | 8 | | 39 | | 3.5 |  |  |
| The first winter Olympics was held in Switzerland | | False | | 8 | | 42 | | 4.15 |  |  |
| The Great fire of London destroyed more than 100 churches | | False | | 10 | | 48 | | 4.5 |  |  |
| The human eyeballs contain 7% salt | | False | | 6 | | 29 | | 3.35 |  |  |
|  | |  | |  | |  | |  |  |  |
| **Statement** | | **Truthfulness** | | **Words** | | **Chars** | | **Rating** | | |
| The human stomach can expand 20 times its normal size | | True | | 10 | | 44 | | 3.85 | | |
| The iris expands three times its normal size when excited | | True | | 10 | | 48 | | 3.8 | |  |
| The longest stick insect in the world is the Palau stick insect | | False | | 12 | | 52 | | 4.1 | |  |
| The Mariner-9 was the first space probe to orbit another planet | | True | | 11 | | 53 | | 4.5 | |  |
| The oldest known infectious disease is rickets | | False | | 7 | | 40 | | 3 | |  |
| The oldest swimming stroke is freestyle | | False | | 6 | | 34 | | 3.6 | |  |
| The photoelectric effect proves the particle behaviour light | | True | | 8 | | 53 | | 4.2 | |  |
| The piano was invented in 1709 | | True | | 6 | | 25 | | 4.3 | |  |
| The shortest war in history lasted 38 hours | | False | | 8 | | 36 | | 3.8 | |  |
| The stethoscope was invented in 1610 | | False | | 6 | | 31 | | 4.45 | |  |
| The sun is one-tenth of the way through its life | | False | | 10 | | 39 | | 4.4 | |  |
| The universe began with a positive net charge | | False | | 8 | | 38 | | 4.25 | |  |
| The world’s oldest museum is in Paris | | False | | 7 | | 31 | | 4.1 | |  |
| The world’s oldest profession is a witch doctor | | True | | 8 | | 40 | | 4.65 | |  |
| There are 36 bones in the human foot | | False | | 8 | | 29 | | 3.7 | |  |
| There are butterflies that smell like vanilla | | False | | 7 | | 39 | | 3.9 | |  |
| There is no proof as to who built the Taj Mahal | | True | | 11 | | 37 | | 2.85 | |  |
| Tiffany and Co. was founded before Italy was a country | | True | | 10 | | 45 | | 4.7 | |  |
| Tigers use black ear spots as identification marks | | False | | 8 | | 43 | | 3.85 | |  |
| Time on Earth is getting faster | | False | | 6 | | 26 | | 4.05 | |  |
| Time stops at the speed of light | | True | | 7 | | 26 | | 4 | |  |
| Toothbrush was invented in 1698 | | False | | 5 | | 27 | | 4.35 | |  |
| Toothbrushes are the oldest device still use today | | False | | 8 | | 43 | | 5 | |  |
| Triskadeccaphobia is the fear of the number 16 | | False | | 8 | | 39 | | 4.6 | |  |
| Trismus is a muscular spasm in the jaw | | True | | 8 | | 31 | | 3.8 | |  |
| Tuberculosis was formally known as assumption | | False | | 6 | | 40 | | 5 | |  |
| Tuberculosis was once known as the white plague | | True | | 8 | | 40 | | 4.1 | |  |
| Uranus is the only planet in our solar system that rolls on its side | | True | | 14 | | 55 | | 4.75 | |  |
| Variola is another name for chickenpox | | False | | 6 | | 33 | | 4.1 | |  |
|  | |  | |  | |  | |  | |  |
| **Statement** | | **Truthfulness** | | **Words** | | **Chars** | | **Rating** | | |
| Vestal virgins were buried alive if they were caught having sex | | True | | 11 | | 53 | | 4.05 | |  |
| Violet light has the most energy | | True | | 6 | | 27 | | 3.9 | |  |
| Virtual images are always inverted | | False | | 5 | | 30 | | 4.4 | |  |
| Windsor Castle was built in 1577 | | False | | 6 | | 27 | | 3.65 | |  |
| Women blink twice as often as men | | True | | 7 | | 27 | | 3.7 | |  |
| Woodpeckers use their tongue to spear food | | True | | 7 | | 36 | | 3.85 | |  |
| Written records of history begin 6,000 years ago | | True | | 8 | | 41 | | 4.05 | |  |
| WWI costed America 10 billion dollars | | False | | 6 | | 32 | | 3.85 | |  |
| WWI is the sixth deadliest conflict in world history | | True | | 9 | | 44 | | 3.7 | |  |
| You can find your occiput on the back on your head | | True | | 11 | | 40 | | 3.45 | |  |
| Zygomatic bone is located in the chin | | False | | 7 | | 31 | | 4.7 | |  |
