# Supplementary figures and images for: Why Do Some Find it Hard to Disagree? An fMRI Study
Source: Front Hum Neurosci. 2016 Jan 29;9:718. doi: 10.3389/fnhum.2015.00718 (PMC4731490; doi:10.3389/fnhum.2015.00718)

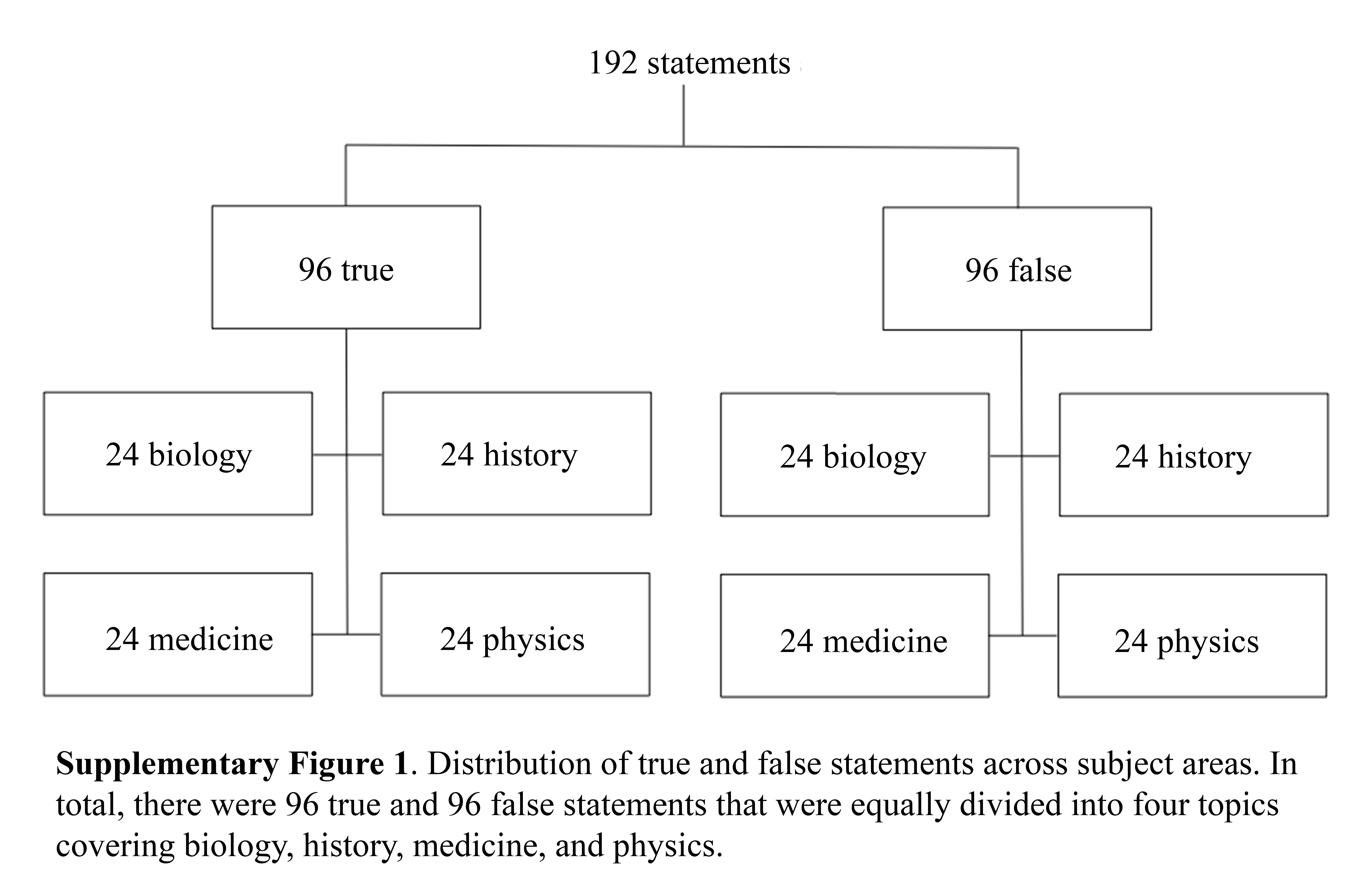

Supplement: Supplementary file 2 [file Image1.TIF]
